# iPSC-Derived MSCs Are a Distinct Entity of MSCs with Higher Therapeutic Potential than Their Donor-Matched Parental MSCs

**DOI:** 10.3390/ijms24010881

**Published:** 2023-01-03

**Authors:** Hae-Ri Lee, Soo Kim, Sungho Shin, Seon-Yeong Jeong, Dae-Won Lee, Sun-Ung Lim, Ji Yeon Kang, Mi-Young Son, Cheolju Lee, Kyung-Rok Yu, Myungshin Kim, Il-Hoan Oh

**Affiliations:** 1 Catholic High-Performance Cell Therapy Center & Department of Medical Life Science, College of Medicine, The Catholic University, Seoul 06591, Republic of Korea; 2 Brexogen Research Center, Brexogen Inc., Songpa-gu, Seoul 05855, Republic of Korea; 3 Chemical & Biological Integrative Research Center, Korea Institute of Science and Technology, Seoul 02792, Republic of Korea; 4 Korean Research Institute for Bioscience and Biotechnology (KRIBB), Daejeon 34141, Republic of Korea; 5 Department of Agricultural Biotechnology, Research Institute of Agriculture and Life Sciences, Seoul National University, Seoul 08826, Republic of Korea; 6 Catholic Genetic Laboratory Center, Department of Laboratory Medicine, Seoul St. Mary’s Hospital, College of Medicine, The Catholic University of Korea, Seoul 06591, Republic of Korea; 7 Regen Innopharm Inc., Seocho-gu, Seoul 06591, Republic of Korea

**Keywords:** iPSC, mesenchymal stromal cells(MSCs), cell therapy, cell autologous, cell differentiation

## Abstract

Mesenchymal stromal cells derived from induced pluripotent stem cells (iMSCs) have been proposed as alternative sources of primary MSCs with various advantages for cell therapeutic trials. However, precise evaluation of the differences between iMSCs and primary MSCs is lacking due to individual variations in the donor cells, which obscure direct comparisons between the two. In this study, we generated donor-matched iMSCs from individual bone marrow-derived MSCs and directly compared their cell-autonomous and paracrine therapeutic effects. We found that the transition from primary MSCs to iMSCs is accompanied by a functional shift towards higher proliferative activity, with variations in differentiation potential in a donor cell-dependent manner. The transition from MSCs to iMSCs was associated with common changes in transcriptomic and proteomic profiles beyond the variations of their individual donors, revealing expression patterns unique for the iMSCs. These iMSC-specific patterns were characterized by a shift in cell fate towards a pericyte-like state and enhanced secretion of paracrine cytokine/growth factors. Accordingly, iMSCs exhibited higher support for the self-renewing expansion of primitive hematopoietic progenitors and more potent immune suppression of allogenic immune responses than MSCs. Our study suggests that iMSCs represent a separate entity of MSCs with unique therapeutic potential distinct from their parental MSCs, but points to the need for iMSC characterization in the individual basis.

## 1. Introduction

Mesenchymal stromal cells (MSCs) are a non-hematopoietic adherent cell population derived from bone marrow (BM), adipose tissue or placental tissue that exhibit multi-lineage differentiation potential towards diverse types of tissues including bone, cartilage, and adipose tissues [1,2,3]. 

Accumulating studies have shown that the primary mode of action for MSCs is the paracrine support of tissue regeneration both by inhibiting apoptosis and fibrosis [4] and by stimulating the regeneration of endogenous stem cells such as hematopoietic stem cells (HSCs), neuronal stem cells, and other tissue-specific endogenous stem cells [5,6]. Accordingly, more than 425 clinical trials have been performed so far using ex vivo expanded MSCs to facilitate the regeneration of injured tissues [7]. However, MSCs obtained from primary tissues exhibit limited proliferating capability and replicative senescence after in vitro culture expansion, hampering wider applications of MSCs in clinical cell therapy trials [8]. Moreover, the functional heterogeneity of MSC populations has been a major problem in standardization for the clinic [9], and the discrepancies generated during in vitro culture of MSCs [10,11,12] pose challenges to maintaining a uniform source. In addition, the quality of MSCs varies widely among donors [13,14,15] making it difficult to produce uniform biological activities in large quantities or to standardize the results of individual clinical and preclinical studies [16]. 

To overcome these issues, recent studies have focused on the generation of mesenchymal stem-like cells from pluripotent stem cells including embryonic stem cells and induced pluripotent stem cells (iPSC) [17]. These iPSC-derived MSCs (iMSCs) have been successfully generated, and exhibited phenotypes and biological functions that mimic MSCs. Thus, iMSCs have been taken as an alternative and more standardized source of MSCs with higher ex vivo expansion potential [18,19,20]. Accordingly, iMSCs have been employed in a wide ranges of cell therapeutic trials including repair of bone defects [21], periodontal regeneration [22], delivery of prodrug-converting enzymes against cancer [23], repair of nerve injuries [24] and other personalized regenerative cell therapies [25]. However, studies have revealed that the iMSCs, despite their MSC-like features, need further characterization due to differences in their cellular properties and differentiation potentials depending on their iPSC source as well as protocols to generate iMSCs [20]. Accordingly, it remains unclear whether the distinct characteristics of iMSCs from parental MSCs originate from the intrinsic changes acquired during the reprogramming process or reflect the differences arising from variations among the individual donor cells.

To address this issue, we generated donor-matched iMSCs from iPSC that had been reprogrammed from the same donor-derived MSCs, and compared the biological characteristics of individual iMSC lines to their parental MSCs. From this study, we show that iMSCs acquire a series of cellular characteristics unique to the reprogrammed MSCs, but these changes still occurred in a donor-dependent manner, raising the importance of donor selection for optimization of iMSCs.

## 2. Result

**Figure 1:**
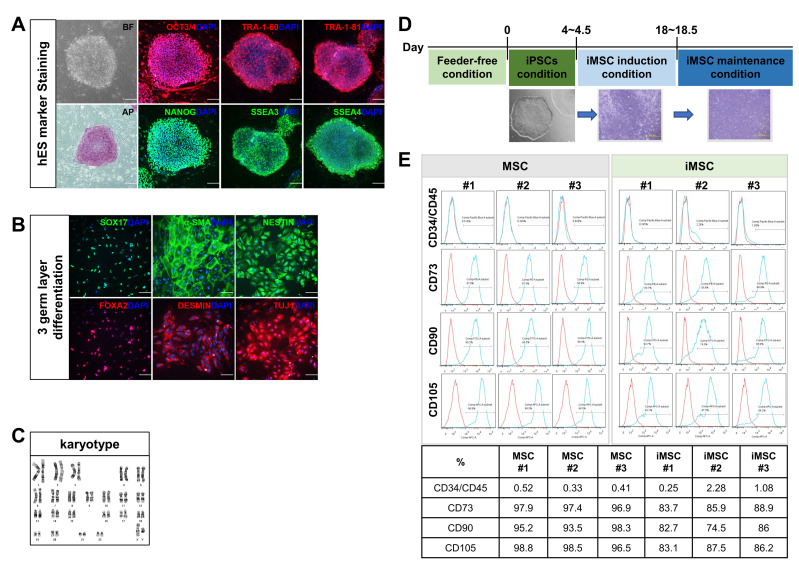
Reprogramming of bone marrow MSCs into induced pluripotent stem cells (iPSCs) and generation of iMSCs from same donor-origin iPSCs. (**A**) Characterization of iPSCs established from primary MSCs. Microscopic examination for morphology in bright field (BF) and expression of indicated pluripotency markers by immunofluorescence analysis. Representative images are shown. (**B**) Immunofluorescence analysis of tri-lineage differentiation potential of iPSCs using endodermal markers (SOX17, FOXA2), mesodermal markers (α-SMA and DESMIN) and ectodermal markers (NESTIN, TUJ1). Nuclei were stained with DAPI. (**C**) Karyotyping of established iPSCs by G-banding analysis confirming normal human karyotypes. (**D**) Schematic diagram of the process for induction of MSCs from iPSC. (**E**) Flow cytometry analysis of MSC markers CD34^−^/CD45^−^, CD73^+^, CD90^+^, CD105^+^ in MSCs and donor-matched iMSCs. The number represents each individual donor.

To compare the cellular characteristics of iMSCs, the mesenchymal stromal cells differentiated from iPSCs, with their autologous MSCs, we first reprogrammed primary MSCs derived from three independent donor’s bone marrow (BM). The BM-derived MSCs were first generated by in vitro adhesion culture and infected with Sendai viral vectors encoding Oct4, Sox2, Klf4 and c-Myc [26,27]. The iPSC lines thus established were verified for expression of pluripotency markers including OCT3/4, TRA-1-60, TRA-1-81, alkaline phosphatase (AP), NANOG, SSEA3 and SSEA4 (Figure 1A). These iPSCs exhibited pluripotency as demonstrated by their potential to differentiate into endosomal tissue (SOX17, FOXA2), mesodermal tissue (alpha-smooth muscle actin; α-SMA and DESMIN), and ectodermal tissue (NESTIN and TUJ1) (Figure 1B). These iPSCs exhibited no chromosomal abnormalities, as determined by cytogenetic analysis (Figure 1C). The iPSCs were then differentiated into MSCs (Figure 1D) and these iPSC-derived MSCs (iMSCs) were found to express the same canonical phenotypic markers as primary MSCs, such as CD34^−^/CD45^−^, CD73^+^, CD90^+^ and CD105^+^ (Figure 1E), confirming the establishment of autologous iMSCs.

**Figure 2:**
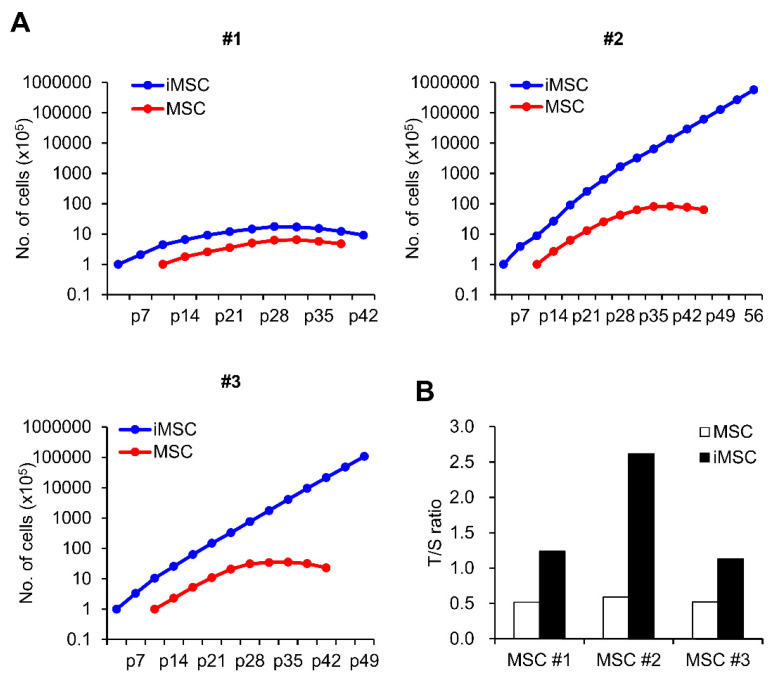
Comparison of proliferation potential between iMSCs and donor-matched primary MSCs. (**A**) Cumulative cell number assay of 3 lines of iMSC and donor-matched primary MSCs. Numbers represent individual donor cell origin. p = passage number. (**B**) Relative telomere length of individual iMSCs in comparison to donor-matched MSCs expressed as a T/S ratio (telomere/single copy) measured by real time PCR.

Next, we examined the biological characteristics of the iMSCs in comparison to their parental donor-matched MSCs. First, we compared their proliferation during in vitro passage subculture. As shown, while variations were observed for individual iMSCs, all three autologous iMSCs exhibited higher proliferative potential than their parental MSCs over 15–17 passages (Figure 2A). Supporting this higher proliferation potential, these iMSCs also exhibited longer telomere lengths compared to their parental MSCs (Figure 2B). However, out of the three autologous iMSCs tested, only two lines (#2, #3 line) exhibited a significant delay in senescence-associated growth arrest, and one of the iMSC lines (#1) whose parental MSCs exhibited low proliferative potential exhibited similarly lower proliferating potential. This indicates that the individual variations of the parental MSCs are preserved during reprogramming and reflected in their subsequent iMSCs (Figure 2A). These results together show that the reprogramming-mediated generation of iMSCs can increase their intrinsic proliferative potential, but these changes are still subjected to variations in the parental MSCs.

**Figure 3:**
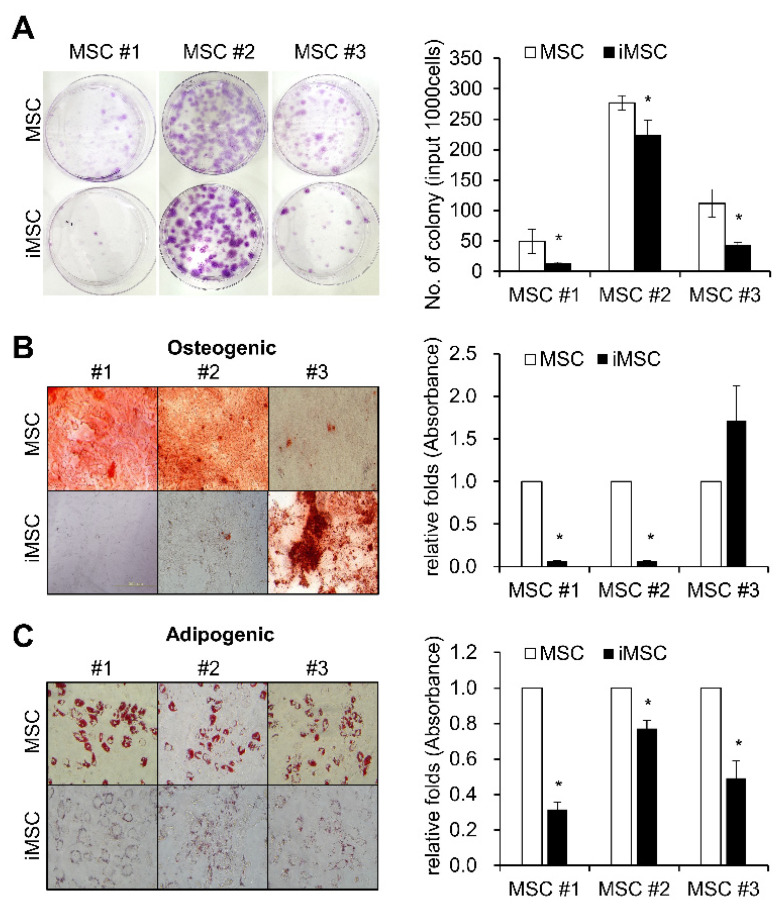
Colonogenic and differentiation potential of iMSCs in comparison with donor-matched MSCs. (**A**) Each individual iMSC line and their parental donor-matched MSCs were subjected to colony formation (CFU-F) assays and stained with crystal violet. Representative images for CFU-F assays (left panel) and their quantification (right) are shown (n = 3, mean ± SEM, *; *p* < 0.05). (**B**) Osteogenic differentiation of MSCs and iMSCs analyzed by Alizarin Red S staining for calcium deposition. Representative images for osteogenic differentiation (left) and quantification as determined by the absorbance at 405nm (right) are shown (n = 3, mean ± SEM, *; *p* < 0.05). (**C**) Adipogenic differentiation of MSCs and iMSCs analyzed by Oil- Red O staining for lipid droplets. Representative images for adipogenic differentiation (left) and the quantification by absorbance at 520nm (right) (n = 3, mean ± SEM, *; *p* < 0.05) are shown.

Studies have shown that MSCs are a heterogeneous cell population exhibiting hierarchical differences among the subsets in their colonogenic or multi-lineage differentiation potentials [28,29]. Since iMSCs are derived from the reprogramming of single iPSC clones and characterized by clonal dominance [30], we were prompted to determine whether the heterogeneity of the parental MSCs is reproduced in the iMSCs. First, we examined the frequency of CFU-F, the colonogenic progenitor population of MSCs among the established iMSCs. As shown, iMSCs exhibited a similar heterogeneity in their colonogenic cell frequencies, and their frequencies tended to be lower than those of the parental MSCs (Figure 3A). These findings show that the heterogeneity of MSCs are reproduced again in iMSCs despite their clonal nature, and that the increased proliferative potential of iMSCs is not correlated to the increased frequency of mesenchymal progenitors, unlike the case for primary MSCs.

Similarly, when we examined the multi-lineage differentiation potential of iMSCs in comparison to the parental MSCs, the three different iMSCs exhibited variable changes in osteogenic differentiation potential (Figure 3B). In contrast, all of the iMSC lines exhibited lower levels of adipogenic differentiation potential than parental MSCs, as determined by reduced deposition of Oil-Red O stain (Figure 3C). These results together show that iMSCs partly reproduce cellular properties in primary MSCs, but also take distinct properties from parental MSCs.

**Figure 4:**
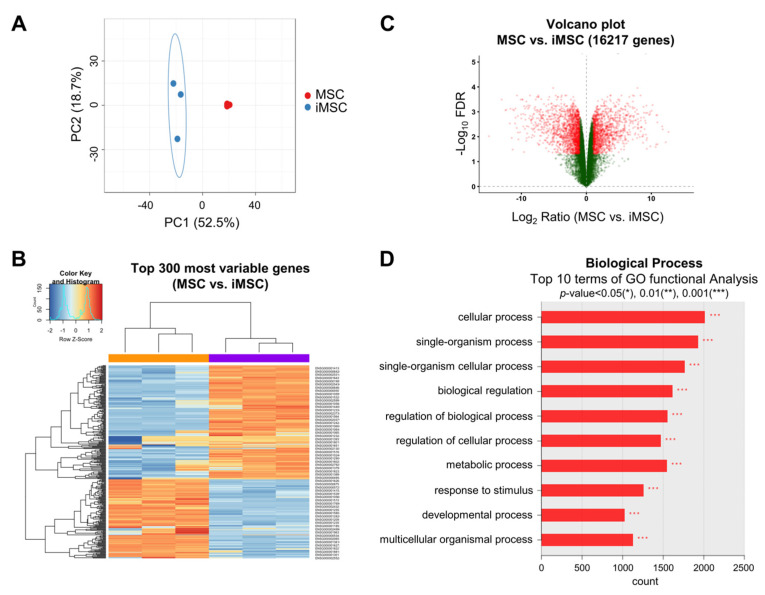
Transcriptomic changes in the transition from primary MSCs to donor-matched iMSCs. (**A**) PCA plot of gene expression data obtained from RNA-seq data for iMSCs (blue dots) and primary MSCs (red dots). (**B**) Heat map of RNA-seq transcriptome analysis for the top 300 most variable genes from iMSCs and donor-matched primary MSCs. (**C**) Volcano plot displaying the log_2_ fold changes (x axis) against the t test-derived −log_10_ FDR (false discovery rate) (y axis) for whole transcriptome differentially expressed between iMSCs and primary MSCs. (**D**) Gene ontology (GO) analysis for differentially expressed genes between iMSCs and MSCs. The top 10 most significant list is shown.

To further explore the distinct cellular characteristics of iMSCs at the molecular level, we next analyzed the transcriptomic differences of iMSCs and their donor-matched parental MSCs. As shown, the transcriptome patterns of iMSCs and parental MSCs were segregated between the two groups, whereas the transcriptomes of individual MSCs were relatively conserved among the iMSCs or parental MSC group (Figure 4A–C). Gene ontology analysis of the transcriptomes revealed gene expression changes in various biological processes controlling the cellular function response to stimuli or developmental processes (Figure 4D). These results indicate that common changes of gene expression patterns controlling cellular functional stage are induced by the reprogramming of MSCs into iMSCs beyond the individual donor variations, thus supporting the notion that iMSCs represent an independent entity in the mesenchymal cell family exhibiting distinctions from their parental MSCs.

**Figure 5:**
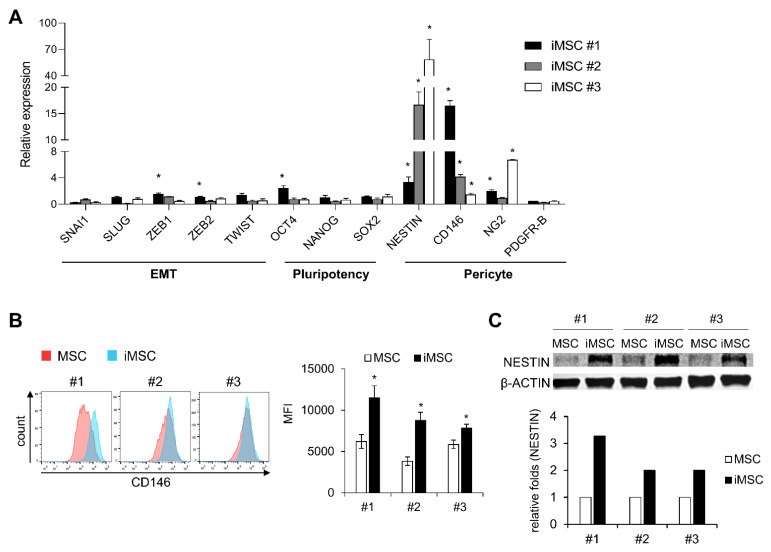
Molecular characterization of iMSCs in comparison with primary MSCs. (**A**) iMSCs and donor-matched primary MSCs were compared for expression of genes involved in the cell fate control of MSCs including genes for EMT, pluripotency and pericyte markers. Shown are normalized expression levels of transcripts in each iMSCs relative to the levels in their donor-matched primary MSCs, analyzed by quantitative RT-PCR (n = 3, mean ± SEM, *; *p* < 0.05). (**B**) Expression levels of human CD146 in iMSCs and their donor-matched primary MSCs. Shown are the representative flow cytometry profiles (left) and quantification of expression levels by mean fluorescent intensity (MFI) (right) (n = 3 for each group, mean ± SEM, *; *p* < 0.05). (**C**) Comparisons for expression levels of human NESTIN between individual iMSCs and their donor-matched parental iMSCs. The expression levels were measured by Western blot. Representative profile (upper) and quantification by image analysis (Image J) (lower) are shown.

To further examine the cell-autonomous differences between parental MSCs and iMSCs, we analyzed expression patterns of genes that characterize the biological properties of MSCs including the epithelial-mesenchymal transition (EMT), pluripotency and markers for pericytes [31,32,33,34,35]. As shown, iMSCs did not exhibit consistent or significant differences in EMT-related genes (*SNAI1*, *SLUG*, *ZEB1*, *ZEB2*, or *TWIST1*) or pluripotency-related genes (*OCT4*, *NANOG*, *SOX2*) (Figure 5A). In contrast, all the iMSCs exhibited significantly increased expression of pericyte markers such as NESTIN and CD146 [32], as defined by transcriptome levels and protein expression levels (Figure 5B,C). These results suggest that reprograming of MSCs into iMSCs causes cell fate changes towards pericyte-like cells.

**Figure 6:**
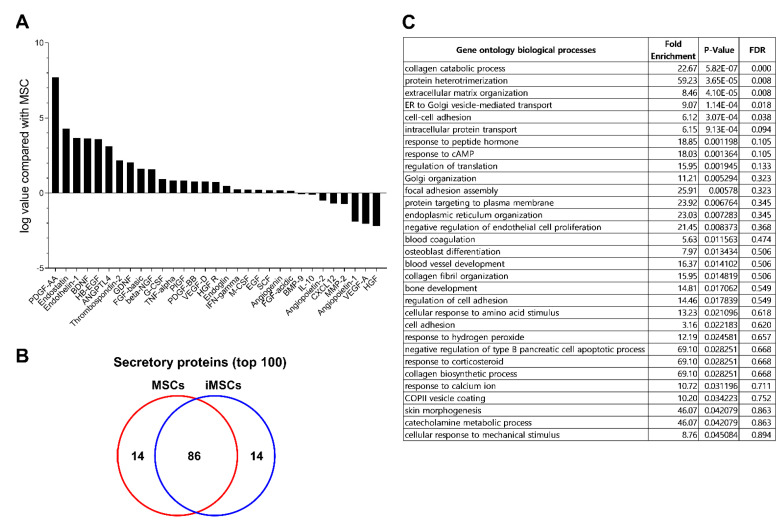
Comparison of secretomes between iMSCs and donor-matched MSCs. (**A**) Cytokine/growth factors in secretomes analyzed by cytokine array. The serum-free supernatant from individual iMSCs and their donor-matched MSCs were harvested and subjected to analysis of cytokine/growth factors using cytokine array blots. Shown are the relative levels of each indicated cytokine/growth factor in the supernatants from iMSCs relative to the levels from their parental MSCs. (**B**) The secretome from each group of MSCs were analyzed by LC-MS/MS. From each secretome, proteins found in at least two independent iMSC or MSC lines were selected and the top 100 secreted proteins of these secretome were identified and compared between the iMSCs and MSCs. (**C**) Gene ontology of secreted proteins up-regulated in iMSCs in comparison to donor-matched MSCs. Shown are the gene clusters of up-regulated proteins in supernatants of iMSCs compared to primary MSCs (*p*-value < 0.05).

Previous studies have shown that pericytes serve as a source of MSCs in multiple types of tissues, and contribute to the microenvironmental niche to support stem cells via paracrine functions [4,5,6]. Therefore, we investigated whether iMSCs exhibit paracrine functions to serve as distinct microenvironment-stimulating cells. For this, we first examined the paracrine secretory factors of iMSCs in comparison to parental MSCs. As shown (Figure 6A), the secretome of iMSCs was distinct from that of MSCs, being mostly enriched with PDGF (platelet derived growth factor), endostatin, endothelin-1, BDNF (brain-derived neurotrophic factor), EGF (epidermal growth factor), and thrombostatin-2 (>2 folds).

To further explore the changes in paracrine function of iMSCs, we examined the whole secretome of iMSCs in comparison to each parental MSC by LC-MS/MS analysis. The serum-free culture supernatants obtained from each type of MSC culture were collected and analyzed for secreted proteins. Overall, 690 proteins (>2 fold differences) were detected from the supernatants. When analyzed for the top 100 secreted proteins, iMSCs and MSCs contained 86 common proteins but 14 of the secretome was unique to each group (Figure 6B). Gene ontology analysis of the distinct secretory proteins showed that the secretome of iMSCs is enriched with factors involved in protein interactions, organization of the extracellular matrix and cell–cell interactions, implicating them in a role in the microenvironment (Figure 6C). Together, these results indicate that iMSCs exhibit distinct paracrine properties from parental MSCs characterized by pericyte-like phenotypes and enhanced microenvironmental support for endogenous stem cells.

**Figure 7:**
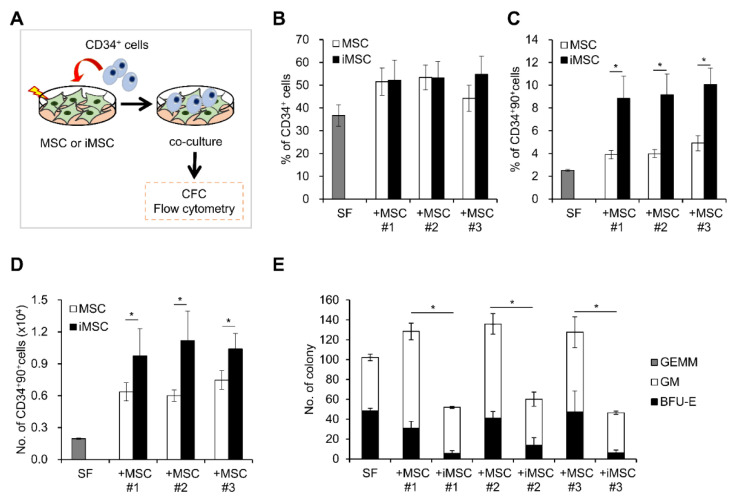
Comparison of HSC supporting activity between iMSCs and donor-matched primary MSCs. (**A**) Schematic illustration of experimental scheme. MSCs from each group were co-cultured with CD34^+^ cells for 4 days and expansion of each indicated subsets of hematopoietic progenitors were analyzed by colony assays or flow cytometry. (**B**) % of CD34^+^ cells in each co-culture condition. (**C**) % of CD34^+^90^+^ cells and (**D**) number of CD34^+^90^+^ cells after co-culture with the iMSCs and donor-matched primary MSCs (n = 6/group, mean ± SEM, *; *p* < 0.05). (**E**) Effect of co-culture on colony forming cells (CFC). Shown are the mean numbers ± SEM of CFCs and their lineages from 300 input CD34^+^ cells after 4 days co-culture with each MSC group (n = 6/group, mean ± SEM, *; *p* < 0.05).

Next, we compared the hematopoietic stem cell (HSC) supporting activities of iMSC and MSCs by co-culturing them with HSCs and measuring maintenance/expansion of hematopoietic progenitors in each co-culture condition. As shown in Figure 7, hematopoietic progenitors co-cultured with iMSCs exhibited comparable frequencies of CD34^+^ cell populations as those in parental MSCs (Figure 7B). However, when examined for more primitive subsets of hematopoietic progenitors (CD34^+^90^+^), iMSCs exhibited significantly higher support for self-renewing expansion of this primitive subset than parental MSCs (Figure 7C,D). In contrast, iMSCs exerted lower support for colony-forming cells (CFC), more down-stream subsets of hematopoietic cells than parental MSCs during the co-culture (Figure 7E). These results indicate that iMSCs exert selectively more support for the primitive subsets of hematopoietic progenitor cells than parental MSCs, but not for the committed colonogenic cells, which is consistent with their pericyte-like phenotypes.

**Figure 8:**
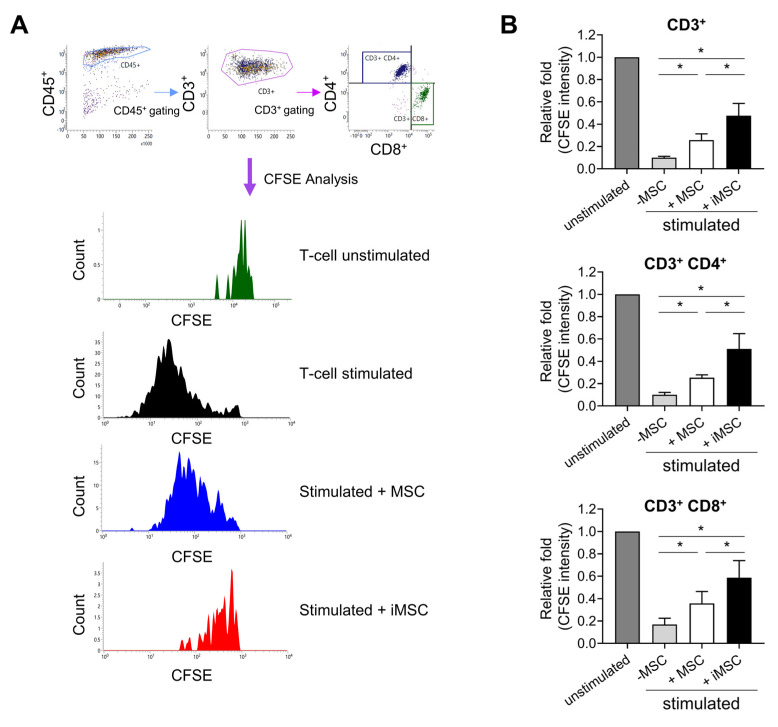
Suppression of the allogenic immune reaction by iMSCs in comparison to donor-matched primary MSCs. (**A**) Representative flow cytometry profiles for analysis of allogenic immune responses by T-cells. Mononuclear cells (MNCs) from umbilical cord blood were stained with CFSE and stimulated with anti-CD3/CD28 microbeads and IL-2 for 6 days in the presence or absence of MSCs or iMSCs. Shown are representative flow cytometry profiles for gating T-cells and their changes in CFSE intensity. (**B**) Quantitative measurement of immune suppressive function of MSCs and iMSCs. The suppression of T-cell proliferation by each type of MSCs was analyzed by the decrease in mean CFSE fluorescence intensity in CD3^+^ or CD3^+^CD4^+^ and CD3^+^CD8^+^ cells. Shown are the mean fluorescence intensity of CFSE in T-cells of each group relative to the intensity in the unstimulated group (one-way ANOVA followed by Tukey’s test, *; *p* < 0.05, *n* = 3).

Notably, MSCs have also been frequently utilized for cell therapeutic trials aimed to suppress allogenic immune reactions in addition to their use for stimulation of endogenous stem cells [36,37]. Therefore, having observed effects on stem cell support, we next compared the immune-modulating activity of iMSCs and donor-matched parental MSCs. Thus, lymphocytes from umbilical cord blood-derived mononuclear cells were stimulated by CD3/CD28 beads and recombinant human IL-2 to trigger allogenic immune reactions in the presence or absence of MSCs or iMSCs. As shown in Figure 8, CFSE-labelled T-lymphocytes were maintained at higher levels in T-cells co-cultured with iMSCs than those with parental MSCs, suggesting less proliferation of T-cells with iMSCs than parental MSCs. The more suppressive effects of iMSCs were observed for CD3^+^, CD3^+^CD4^+^ and CD3^+^CD8^+^ cells, indicating more potent suppressive effects of iMSCs than parental MSCs on the allogenic stimulation of T-cell subsets.

These results show that iMSCs can exert more potent immune suppression than parental MSCs, suggestive of advantages in trials for immune-modulating therapeutic applications, in addition to advantages for support for endogenous stem cells.

## 3. Discussion

The cell therapeutic applications of MSCs have been expanding over the decades in a variety of clinical settings, but the limitation of the source for MSCs has been a major hurdle. The large-scale application of primary MSCs still presents challenges due to the limited numbers of cells that can be obtained from the donor. Moreover, primary MSCs exhibit a decline in proliferative activity with increasing age and underlying disorders [15,38]. Similarly, culture expansion of MSCs to large numbers of cells is limited by continuous gene expression changes and replicative senescence [11]. Derivation of MSCs from highly expandable iPSCs could overcome these limits, providing the possibility for larger expansion of MSCs as a source of expandable banking of standardized cells [23], which can also provide the opportunity for standardization of therapeutic effects of MSCs. Moreover, iPSC-derived MSCs were suggested to be partially rejuvenated in terms of DNA methylation patterns [18], exhibiting several distinctive features in their regenerative potential, including for heart, osteogenic tissues or kidney, tumor trophism and immune suppressive function [21,39,40,41,42,43,44,45,46]. However, despite these notions, the precise differences in cellular properties of iMSCs from parental MSCs, and the origin of their distinctions has not been clearly understood. While it is possible that the distinctive features originate from the intrinsic cellular reprogramming process, it was also possible that part of those observed differences in iMSCs could have been caused by individual donor-based differences of the MSCs.

To address these issues, in this study we generated donor-matched iMSCs from multiple donor-derived MSCs and compared various cellular elements of MSCs in a donor-matched comparison. Interestingly, our study shows that not all of the cellular differences previously thought to be distinctive features of iMSCs were intrinsic legitimate properties of reprogrammed iMSCs. For example, while iMSCs are frequently depicted as cells with higher proliferative potential and delayed senescent growth arrest during culture [20], our study shows that those differences were still dependent on the initial properties of the parental MSCs. Consistent with our observations, previous studies showed that iPSC-derived MSCs also underwent senescence after ex vivo culture or increasing age of donor cells [38,46]. Taking these findings together, our study suggests that iPSCs exhibit variations in their cellular properties influenced by the initial properties of their parental cells, thus suggesting caution in the interpretation on the innate properties of iPSC.

Similarly, the changes in the multi-lineage differentiation potential of MSCs [2,3] was not consistent in our multi-donor comparison, displaying variations for each iMSC line. These variations in osteogenic potential were similarly reported in a previous study, where iMSCs were shown to retain their osteogenic differentiation potential with larger variations in their ability to reconstitute osteogenic tissues [47]. Another study showed that iMSCs could facilitate repair of bone defects with higher osteogenic potential than MSCs although it failed to induce ectopic bone formation [21]. The precise reason for these discrepancies remains unclear. However, our observations for individual variations of osteogenic differentiation raise the possibility that individual variations of MSC or iMSCs in each study model could be a factor underlying different outcomes. Supporting this possibility, one study based on donor matching comparisons of MSCs and iMSCs reported a decreased response of iMSCs to differentiation signals, thus revealing the variable differentiation potential in iMSCs [48].

However, it is noteworthy that iMSC itself can exhibit variation depending on the process or source of the cells for their production. For example, iMSCs exhibited substantial differences in terms of tri-lineage potential and gene expression patterns compared to primary MSCs even in cells derived from the same donor depending on the methods for generation of iMSCs [48]. Likewise, iMSCs generated from iPS cell lines from different tissue origins exhibited variability in their osteogenic differentiation patterns and therapeutic potential [39,49], thus indicating that the nature of the parental cells and/or generation protocols could also contribute to variations in the biological characteristics of iMSCs.

Interestingly, heterogeneity of iMSCs in their clonogenic potential provided insights into the origin of heterogeneity in MSCs. Studies on primary MSCs in bone marrow have shown that colonogenic MSCs (CFU-F) originate from primitive subsets of the mesenchymal cell population with specific phenotypic markers such as nestin, leptin receptors, prx-1, PDGFR-alpha, or CD51 [50,51,52,53]. Given that the iMSCs are derived from clonal differentiation of iPSCs with clonal dominance [30], our finding that only subsets of these iMSCs retain clonogenic potential suggest that the generation of heterogeneity in MSC populations should be an intrinsic property of the MSC population for ex vivo cultured MSCs. Further studies are warranted to test this possibility in various in vivo and in vitro models for the generation of MSC populations.

Of note, the transition from MSCs to iMSCs was accompanied by significant gene expression changes both in the genome-wide transcriptomic and proteomic profiles, while maintaining conserved gene expression patterns among the individual groups. This finding raises the possibility that unique gene expression patterns are acquired in iMSCs during the transition from primary MSCs, thus resulting in common differences between MSCs and iMSCs beyond the variations among individual donors.

The precise nature of these changes in the gene expression programs remains unclear. However, we found that iMSCs exhibit significantly higher-level induction of pericyte markers such as NESTIN and CD146, which have been used to identify the subsets of MSCs endowed with the ability to support endogenous stem cells [50]. In addition, comparisons of secretomes from MSCs and iMSCs showed that iMSCs secrete significantly higher levels of cytokines/growth factors and proteins involved in cellular interactions with ECM or cell–cell interactions, suggesting roles in enhanced paracrine function.

Consistent with this possibility, iMSCs exhibited higher support for the self-renewing expansion of primitive hematopoietic progenitors, as defined by numbers of long-term in vivo repopulating HSC subsets (CD34^+^90^+^) in the co-culture [54]. However, it should be mentioned that another study reported a lower supportive activity of iMSCs than primary MSCs, particularly for long-term culture initiating cells [44]. While these discrepancies should also be interpreted in the context of our donor-matched comparative study, it is also possible that measurement of distinct subsets of hematopoietic progenitors could also have attributed to the differences, given previous reports that subsets supporting long-term culture could be distinct from subsets supporting BM repopulating cells [55,56]. Further studies are warranted to evaluate the influence of iMSCs on in vivo hematopoietic reconstitution in comparison to primary MSCs.

Interestingly, iMSCs exerted more potent immune suppressive effects on allogenic immune stimulation, in the suppression of both CD4^+^ and CD8^+^ cell proliferation. Consistent with our observations, it was shown that iPSC-derived MSCs were insensitive to proinflammatory IFN-γ-induced HLA-II expression [43] and exhibited higher potency in the inhibition of NK-cell proliferation and cytolytic activities compared to parental BM MSCs [57]. Accordingly, these distinct immune-suppressive activities by iMSCs could be advantageous in cell therapeutic trials evaluating immune-modulating effects.

In summary, our study on the donor-matched comparisons of iMSCs and primary MSCs revealed that iMSCs might represent another alternative source for MSCs with multiple functional distinctions from their parental MSCs, including higher proliferation potential, enhanced paracrine functions for stem cell support as well as more potent immune regulatory effects. However, our study also shows that significant functional heterogeneities exist among the iMSCs, being influenced by intrinsic properties of the parental cells, indicating the importance of donor screening and standardization of iMSCs on an individual basis.

## 4. Materials and methods

### 4.1. Human Cells

Human MSCs were separated from Bone marrows (BMs) of healthy donors under informed consent and approval by Institutional Review Board of the Catholic University of Korea (MC19SNSI0059, 09-05-2019). Human MSCs cultures were established from BM mononuclear cells and passaged in low-glucose Dulbecco’s modified Eagle’s medium (DMEM; Hyclone Laboratories Inc, Schenectady, NY, USA) containing 15% fetal bovine serum (FBS; Hyclone, Logan, UT, USA), L-glutamine, 100 U/mL penicillin, and 100 mg/mL streptomycin in a humidified 5% CO2 at 37 °C. Human CD34^+^ cells were isolated from umbilical cord blood (UCB) using immunomagnetic columns and Dynabeads (Invitrogen, Carlsbad, CA, USA) and pooled together for experiments. UCB was obtained directly from 7 healthy pregnant woman donors under written informed consent. The consent form and all experiments in this study were approved by the Institutional Review Board of the Catholic University of Korea (MC19TNSI0012, 15-03-2019).

### 4.2. Human iPSC Generation and Culture

To generate integration-free iPSC lines, human MSCs derived from healthy donors were electroporated with Episomal iPSC Reprogramming Vectors (Invitrogen, Carlsbad, CA, USA) using the Neon Transfection System (Invitrogen) at a pulse voltage of 1650 V, a pulse width of 10 ms and a pulse number of 3 according to the manufacturer’s instructions. The electroporated cells were evenly plated on two sets of 35 mm dishes. At 5 days after electroporation, the transfected cells were seeded onto Matrigel (Corning, NY, USA)-coated 6-well plate. The cells were fed daily with mTeSR1 medium (Stem Cell Technologies, Vancouver, BC, Canada). To increase reprogramming efficiency, nicotinamide (1 mM) was supplemented during the reprogramming process. After 14 or 21 days, human ESC-like iPSC colonies were picked, subsequently passaged and expanded for further characterization. Then, MSC-specific human iPSC lines were grown on Matrigel in mTeSR2 (Stem Cell Technologies).

### 4.3. In Vitro Differentiation of iPSC into Specific Lineages

hiPSCs were differentiated into EB as previously described [58]. Briefly, the clumps of hiPSCs were cultured in suspension in EB medium supplemented with Knockout DMEM (Invitrogen), 20% FBS (Invitrogen), 1% non-essential amino acids, 1 mM L-glutamine, 0.1 mM β-mercaptoethanol, and 1% penicillin/streptomycin. The culture medium was refreshed every 2 days. For spontaneous in vitro differentiation of hEBs, the cell aggregates were seeded onto gelatin-coated plates and cultured in hEB medium for an additional 10 days. The medium was changed every two days.

Thus differentiated hEBs were subjected to differentiation into specific lineages as previously described [58]. Briefly, for ectoderm differentiation (neurons, oligodendrocytes, astrocytes), the hEBs were cultured in neural stem spheres (NSSs) culture medium (DMEM/F12 supplemented with 1× N2/B27 (Invitrogen), 20 ng/mL epidermal growth factor (Invitrogen), 20 ng/mL bFGF, 10 ng/mL leukemia inhibitory factor (Sigma) and 100 U/mL penicillin-streptomycin) differentiated into NSSs. The NSSs were sub-cultured every week using a McIlwain tissue chopper (Mickle Engineering, Gomshall, Surrey, UK), and the medium was refreshed every 2 days. For terminal differentiation into neurons, oligodendrocytes, astrocytes, the NSSs were allowed to attach to Matrigel-coated coverslips and maintained in the same NSS medium without growth factors for 3–4 weeks.

For mesoderm differentiation (osteoblasts), the hEBs were plated in Matrigel-coated dishes and cultured in medium containing osteogenic supplements and 0.1 mM L-ascorbic acid (Sigma), 10 mM β-glycerophosphate (Sigma), and 0.1 mM dexamethasone (Sigma) for 3–4 weeks. For mesoderm differentiation (cardiomyocytes), the hEBs were plated in gelatin-coated cell culture dishes in differentiation medium consisting of Knockout DMEM (Invitrogen), 20% FBS (Invitrogen), 1% non-essential amino acids, 1 mM L-glutamine, 0.1 mM β-mercaptoethanol, and 1% penicillin/streptomycin for 3 weeks.

For endoderm differentiation (endothelial cells), the following protocol was used: hEBs were maintained in hEB medium supplemented with 20 ng/mL BMP4 (R&D Systems, Minneapolis, MN, USA) (removed on day 7); on day 1, the medium was supplemented with 10 ng/mL activin A (Peprotech) (removed on day 4); on day 2, the medium was supplemented with 8 ng/mL bFGF (R&D Systems) (for the duration of the culture); on day 4, the EBs were transferred to adherent conditions in Matrigel-coated plates, and the medium was supplemented with 25 ng/mL VEGF-A (Peprotech) (for the duration of the culture); finally, on day 7, 10 µM SB431542 (Sigma) was added to the culture followed by an incubation for 7 days.

### 4.4. Alkaline Phosphatase and Immunofluorescence Staining

For alkaline phosphatase staining, iPSCs were fixed in a citrate–acetone–formaldehyde solution and then stained with alkaline phosphatase staining solution (Naphthol/fast red violet, Sigma). Cell images were captured using an Olympus microscope (IX51, Olympus, Tokyo, Japan).

For immunofluorescence staining, cells were fixed in 4% formaldehyde and then permeabilized with phosphate-buffered saline containing 0.1% Triton X-100 (Sigma). After blocking with 3% bovine serum albumin (Sigma), cells were incubated with the following primary antibodies for human embryonic stem cells (hES) markers: OCT3/4 (Santa Cruz Biotechnology, Santa Cruz, CA, USA), TRA-1-60 (Chemicon, Santa Cruz, CA, USA), TRA-1-81 (Chemicon), NANOG (Santa Cruz Biotechnology), SSEA3 (R&D Systems, Minneapolis, MN, USA), and SSEA4 (R&D Systems). Primary antibodies for three germ layer differentiation: SOX17 (R&D Systems), α-smooth muscle actin (Sigma-Aldrich, Carlsbad, CA, USA, A5228), NESTIN (Chemicon), FOXA2 (Abcam, Cambridge, MA, USA), DESMIN (Chemicon) and TUJ1 (Covance, Munich, Germany). DAPI (4′,6-diamidino-2-phenylindole, KPL) was used for nuclear counterstaining. Chamber slides were observed with an Axiovert 200M microscope (Carl Zeiss, Gottingen, Germany) or an Olympus microscope (Olympus).

### 4.5. Karyotype Analysis

G-banding karyotype analysis of human MSC-derived iPSC lines was performed by GenDix, Inc. (Seoul, Republic of Korea).

### 4.6. iPSC-Derived MSC Generation and Culture

To generate MSCs from human MSC-specific iPSC lines, bulk cultures were seeded in mTeSR2 medium (as per the manufacturer’s instructions) on Matrigel-coated dishes. When iPSCs formed large colonies at high confluence, the medium was changed to MSC differentiation media. For differentiation of iPSCs to MSCs, Dulbecco’s Modified Eagle Medium (DMEM) (Hyclone, USA) with bFGF (peprotech) 5 ng/mL, 0.1 mM NEAA, 1% Glutamax, 1% antibiotics (Gibco) and 10μM SB431542 in DMSO (Sigma) was replaced every 2 days for 14 days, with cells then passaged to a single cell suspension using Trypsin/EDTA (Invitrogen). For maintenance of iPSC-derived MSCs were cultured in Dulbecco’s Modified Eagle Medium (DMEM) with 15% FBS (Hyclone), L-glutamine, 100 U/mL penicillin, and 100 mg/mL streptomycin onto 0.1% gelatin (Sigama)-coated tissue culture dishes.

### 4.7. Co-Culture

For co-culture assays, MSCs were irradiated with 15 Gy of ^137^Cs γ-rays (Gammacell 1000; MDS Nordion, Ottawa, ON, Canada) 18–24 h prior to co-culture with CD34^+^ cells. Purified CD34^+^ cells co-cultured with MSCs for 4 days in DMEM supplemented with FBS in the presence of a cytokine mixture (20 ng/mL human SCF; 20 ng/mL human Flt3L (ProSpec-Tany TechnoGene Ltd.), 4 ng/mL human IL3, IL6 (R&D Systems, Minneapolis, MN), and 4 ng/mL human G-CSF (ProSpec) supplemented with 10^−6^ M hydrocortisone (Sigma-Aldrich, St Louis, MO). Controls were performed by seeding CD34^+^ cells in suspension culture without MSCs.

### 4.8. Flow Cytometry

MSCs were analyzed by flow cytometry (LSRll) using the following antibodies: anti-human CD34-BV421 (1:20), CD45-BV421 (1:20), CD73-PE (1:50), CD90-FITC (1:100), CD105-APC (1:20), and CD146-PEcy7 (1:50) (BD Pharmingen, USA). For analysis of co-cultures, CD45-APC (1:50), CD34-PE (1:25), and CD90-FITC (1:100) (BD PharMingen) antibodies were used. Cells were stained for 30 min on ice after adding the optimal concentration of antibody.

### 4.9. Telomere Length Analysis

MSCs telomere lengths were measured with a qPCR-based technique that compares telomere repeat sequence copy number to single-copy gene (36B4) copy number. The telomere-specific primers (forward: 5′GGTTTGTTTGGGTTTGGGTTTGGGTTTGGGTTTGGGTT3′; reverse: 5′GGCTTGCCTTACCCTTACCCTTACCCTTACCCTTACCCT3′) and the 36b4 primers (forward: 5′CAGCAAGTGGGAAGGTGTAATCC3′; reverse: 5′CCCATTCTATCATCAACGGGTACAA3′) were prepared. All PCRs were performed on the Rotor-Gene Q real-time instrument (QIAGEN, Hilden, Germany). The thermal cycling profile for both amplicons began with 95 °C incubation for 10 min. For telomere PCR, there followed 25 cycles of 95 °C for 15 s, 58 °C for 1 min. For 36B4 PCR, there followed 30 cycles of 95 °C for 15 s, 58 °C for 1 min. Rotor-Gene Q software 2.0.2 was then used to generate the standard curve for each plate and to determine the dilution factors of standards corresponding to the T (telomere) and S (single copy; b-globin) amounts in each sample. Relative T/S ratios reflect relative length differences in telomeric DNA.

### 4.10. CFU-F Assay and Differentiation of MSCs

For colony forming unit-fibroblast (CFU-F) assays, MSCs were plated 1000 cells in 100mm tissue culture dishes. MSCs and iMSCs were maintained in DMEM containing 15% FBS, L-glutamine, 100 U/mL penicillin, and 100 mg/mL streptomycin in a humidified 5% CO2 at 37 °C. 14 days after plating, the cells were fixed with methanol and then stained with crystal violet (Sigma) for visualization.

Osteogenic differentiation was induced by StemPro osteogenesis differentiation media (Thermo Fisher). MSCs or iMSCs exchanged differentiation medium every 3 days. After 8 days, the mineralization of the extracellular matrix was determined by Alizarin Red S (Sigma) staining, and extraction with 10% cetylpyridinium chloride (Sigma) followed by measurement of the absorbance at 405 nm.

For adipogenic differentiation, confluent cells were incubated in StemPro adipogenesis differentiation media (Thermo Fisher). MSCs or iMSCs exchanged differentiation medium every 3 days. After 10 days, cells were fixed with propylene glycol (Sigma) and stained with Oil Red O (Sigma) to visualize lipid droplets. The numbers of lipid droplets were subsequently determined by dye extraction using 4% Nonidet P40 (Sigma) in isopropyl alcohol (Sigma) followed by spectrophotometry at 520 nm.

### 4.11. Magnetic Bead-Based Multiplex Assay for Cytokine/Growth Factor

To quantitatively measure the cytokine/growth factors in the supernatants of iMSC or MSC, cells were plated at a density of 1 × 10^5^/well in six-well plates and allowed to adhere overnight. After replacing the medium with serum-free medium (1 mL), cells were further cultured for 48 h. The medium was then collected and analyzed with Human Magnetic Luminex^®^ Screening Assay (R&D Systems) following the manufacturer’s instructions.

### 4.12. Western Blot

Expression of pericyte markers (NESTIN) was analyzed by Western blots, MSC and iMSC lysates were subjected to gel electrophoresis (SDS-PAGE), transferred to membranes, and incubated with primary antibodies against NESTIN (Abcam). The membranes were then incubated with horseradish peroxidase-conjugated secondary antibodies, and proteins were visualized using a chemiluminescence substrate (Thermo Scientific, IL, USA). The Luminescent Image Analysis System (LAS-4000; Fuji film, Tokyo, Japan) with quantification using Image J software.

### 4.13. CFSE (Carboxyfluorescein Succinimidyl Ester) Proliferation Assay

For T-cell proliferation assays, CFSE-labeled mononuclear cells (MNCs) were co-cultured with growth arrested MSCs in RPMI 1640 (Rosewell-Park Memorial Institute) medium supplemented with 10% FBS for 6 days, in the presence of anti-CD3/CD28 microbeads (Gibco) and recombinant human IL-2 (30 U/mL, PeproTech). CFSE-labeled MNCs were prepared by isolation of human MNCs from UCB by Ficoll-Paque (Ficoll-Paque PLUS, GE Healthcare, Chicago, IL, USA) gradient, followed by labeling with CFSE using a CellTrace CFSE Cell Proliferation Kit (2 μM, Thermo Fisher) according to the manufacturer’s instructions. The growth arrested MSCs were prepared by treatment of MSCs with 10 μg/mL of mitomycin C (MMC, Sigma) for 1 h, trypsin detachment, then 2 times washing with PBS before co-culture with CFSE-labeled MNCs.

Six days after co-culture, the cells were stained with fluorescence-labeled human antibodies against CD45-BV421, CD3-PE, CD4-APC, and CD8-BV605 (BD), and changes of the CFSE intensity in the T cell population (gated for CD3, CD4, CD8) were measured by flow cytometry for T cell proliferation.

### 4.14. Real Time-PCR

Total RNA was extracted from MSCs using Trizol (Invitrogen). cDNA was synthesized from 1μg of total RNA with reverse transcriptase (Invitrogen). Real-time quantitative PCR (RT-qPCR) was performed with SYBR premix Ex taq (Takara, Japan). The threshold cycle (Ct) value for each gene was normalized to the Ct value of hHPRT. The relative mRNA expression was calculated by using the formula; 2^−ΔΔCt^, where ΔCt = Ct_sample_ − Ct_HPRT_ and ΔΔCt = Δ Ct_sample_ − ΔCt_reference_ group.

### 4.15. mRNA-seq Data

Whole-transcriptomes of iMSC or MSCs were analyzed by mRNA-seq as previously described [59,60].Briefly, paired sequencing reads were aligned onto the reference human genome sequences (HISAT v 2.1.0). The estimation of gene expression (fragments per kilobase of exon per million fragments mapped [FPKM]) was performed using CuffLink software. The relative abundances of gene were measured in Read Count using StringTie. Statistical significance of the differential expression data was determined using nbinomWaldTest using DESeq2 and fold change in which the null hypothesis was that no difference exists among groups. False discovery rate (FDR) was controlled by adjusting *p* value using Benjamini-Hochberg algorithm. The GO gene sets that showed significant enrichment (nominal *p* < 0.05) were selected with leading edge genes.

### 4.16. Preparation of the Secretome

MSCs and iMSCs were cultured until 70% confluence, washed three times with serum-free media (SFM) omitting phenol red, and incubated in that same SFM for an additional 24 h. The protease inhibitors phenylmethylsulfonyl fluoride (Sigma) and ethylenediaminetetraacetic acid (USB, Cleveland, OH, USA) were added to the conditioned medium at final concentrations of 2 mM and 1 mM, respectively. Cell debris was removed by centrifugation (400× *g*, 20 min, 4 °C) and sterile filtration (pore size: 0.22 μm, Millipore, Billerica, MA, USA).

### 4.17. Mass Spectrometric (MS) Analysis of Secreted Proteins and Bioinformatics Analysis of MS Data

The collected secretome samples (100 μg protein) were analyzed by nano-flow liquid chromatography tandem mass spectrometry (LC–MS/MS) on an LTQ-Orbitrap XL mass spectrometer (Thermo Fisher Scientific) after tryptic digestion. Each biological replicate of the secretome was analyzed in a single LC–MS/MS run.

The mass spectrometric data were loaded on the Proteome Discoverer (ver2.2.0.388) software, and label-free quantification (LFQ) was performed to compare the expression levels of each individual protein between replicates and between different MSCs. We used the human UniProtKB database (released in June 2020) with the FBS protein list added for the MS data search to exclude suspected proteins affected by FBS by potential contamination of the FBS. Only the proteins confirmed as true secretory proteins by either SignalP, SecretomeP (score ≥ 0.5) or TMHMM were used for the next step of gene ontology (GO) analysis. GO terms were analyzed using the algorithm of the Database for Annotation, Visualization, and Integrated. Discovery (DAVID) tools (https://david.ncifcrf.gov/, accessed on 19 March 2021).

## Data Availability

Not applicable.
